# Supplementary material for: Population-specific association of Clock gene polymorphism with annual cycle timing in stonechats
Source: Sci Rep. 2022 May 13;12:7947. doi: 10.1038/s41598-022-11158-z (PMC9106710; doi:10.1038/s41598-022-11158-z)
Supplement: Supplementary file 1 — Supplementary Information. [file 41598_2022_11158_MOESM1_ESM.pdf]

Supplementary Material

Population-specific association of *Clock* gene polymorphism with annual cycle timing in stonechats

Hannah Justen*[1,2], Timo Hasselmann[1,3], Juan Carlos Illera[4], Kira Delmore[1,2], David Serrano[5], Heiner Flinks[6], Masayuki Senzaki[7], Kazuhiro Kawamura[8,9], Barbara Helm[10,11] and Miriam Liedvogel*[1,12]

[1] MPRG Behavioural Genomics, Max Planck Institute for Evolutionary Biology, 24306 Plön, Germany; [2] Department of Biology, Texas A&M University, 3258 TAMU, College Station, TX, 77843, US; [3] Department of Animal Physiology, University of Cologne, Cologne, Germany; [4] Biodiversity Research Institute (CSIC-Oviedo University-Principality of Asturias), Oviedo University, Oviedo, Spain; [5] Department of Conservation Biology; Estación Biológica de Doñana (EBD-CSIC); Avda Américo Vespucio 26; 41092 Sevilla, Spain; [6] Wagnerstr. 19, D-46354 Suedlohn, Germany; [7] Environmental Earth Science, Hokkaido University, Sapporo, Hokkaido, Japan; [8] Graduate School of Agriculture, Hokkaido University, Kitaku Kita 9 Nishi 9, Sapporo, Hokkaido, Japan; [9] Department of Wildlife Biology, Forestry and Forest Products Research Institute, Matsunosato 1, Tsukuba, Ibaraki 305-8687, Japan; [10] Department of Bird Migration, Swiss Ornithological Institute, Seerose 1, 6204, Sempach, Switzerland; [11] Institute of Biodiversity, Animal Health and Comparative Medicine, University of Glasgow, Glasgow, UK; [12] Institute of Avian Research 'Vogelwarte Helgoland', 26386, Wilhelmshaven, Germany

Table S1. Breeding latitude, migration distance [km], *Clk* poly-Q repeat length frequencies ($Q_{8\text{-}15}$) and gene diversity (GD) of nine stonechat (*Saxicola spp.*) populations listed by increasing latitude. Gene diversity was calculated with a subset of the data including 518 birds captured in the wild (i.e. excluding captive-bred individuals).

Populations	n	Latitude	Distance	Q8	Q9	Q10	Q11	Q12	Q13	Q14	Q15	GD
Kenya	95	0°14’S	0	0	0.005	0	0.089	0.032	0.858	0.016	0	0.256
Tanzania	44	3°5’S	0	0	0.011	0.023	0.045	0.023	0.864	0.034	0	0.253
Canary	61	28°46’N	0	0	0	0	0.008	0	0.008	0.984	0	0.033
Spain	93	37°39’N	0	0.089	0	0	0.258	0	0.21	0.511	0	0.631
Japan	11	43.6°N	4900	0.032	0	0	0.273	0	0.546	0.182	0	0.609
Austria	64	48°14’N	1700	0.023	0	0	0.164	0.016	0.156	0.641	0	0.541
Germany	47	51°47’N	1700	0.011	0.021	0	0.160	0.021	0.213	0.564	0.011	0.617
Kazakhstan	60	51.5°N	2600	0	0	0.017	0.208	0	0.583	0.050	0.142	0.597
Ireland	106	52°N	1200	0	0.005	0	0.104	0.038	0.462	0.392	0	0.623

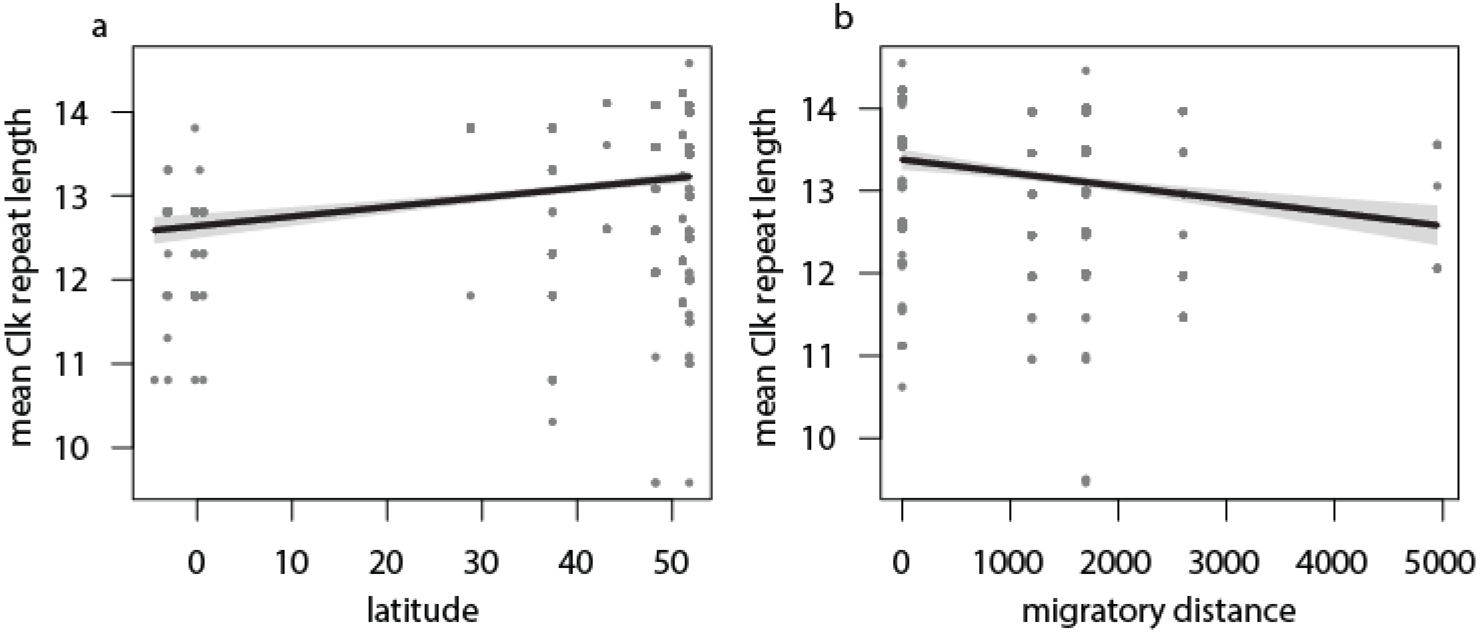

Figure S1. Results from linear model investigating breeding latitude (a) and migratory distance (b) of nine stonechat populations in relationship to *Clk* gene mean repeat length (including origin as a random effect). The model shows that mean repeat length of individuals increases at higher latitudes and decreases with longer migratory distance ($p < 0.05$).
